# An Analysis of the Connectedness to Nature Scale Based on Item Response Theory

**DOI:** 10.3389/fpsyg.2017.01330

**Published:** 2017-08-04

**Authors:** Laura Pasca, Juan I. Aragonés, María T. Coello

**Affiliations:** Faculty of Psychology, Complutense University of Madrid Madrid, Spain

**Keywords:** connectedness, Item Response Theory, CNS, nature, inclusion of nature in self

## Abstract

The Connectedness to Nature Scale (CNS) is used as a measure of the subjective cognitive connection between individuals and nature. However, to date, it has not been analyzed at the item level to confirm its quality. In the present study, we conduct such an analysis based on Item Response Theory. We employed data from previous studies using the Spanish-language version of the CNS, analyzing a sample of 1008 participants. The results show that seven items presented appropriate indices of discrimination and difficulty, in addition to a good fit. The remaining six have inadequate discrimination indices and do not present a good fit. A second study with 321 participants shows that the seven-item scale has adequate levels of reliability and validity. Therefore, it would be appropriate to use a reduced version of the scale after eliminating the items that display inappropriate behavior, since they may interfere with research results on connectedness to nature.

## Introduction

In the field of environmental concerns, the relationship between the *self* and nature has been a subject of increasing study in recent years. Ideas in this regard have been grouped under the label of environmental connectedness (Beery and Wolf-Watz, 2014). Human beings depend both physically and emotionally on nature, and at the same time, nature depends on human beings (Davis et al., 2009). The complexity of this relationship emerges when it is operationalized, and so researchers have developed different concepts in which this link always has an underlying role, with emphasis placed on different aspects. Accordingly, in research one encounters terms such as: “emotional affinity toward nature” (Kals et al., 1999), “Inclusion of Nature of Self” (Schultz, 2001), “environmental identity” (Clayton, 2003), “connectedness to nature” (Mayer and Frantz, 2004), “connectivity with nature” (Dutcher et al., 2007), “commitment to the environment” (Davis et al., 2009), “Nature Relatedness” (Nisbet et al., 2009) and “love and care for nature” (Perkins, 2010). However, despite the different concepts and measures, these are all expressions of the same construct: a subjective connection to nature (Tam, 2013; Capaldi et al., 2014). In fact, Tam (2013) found that the different concepts formed a single latent construct, but upon comparing the correlations of each measure with the different criterion variables traditionally related to connectedness, it was observed that the magnitude of these correlations is not equal.

Relations between the *self* and nature are not only biophysical in nature. Rather, other dimensions corresponding to cultural and spiritual development can also be contemplated. For some authors (Dutcher et al., 2007), relations not only refer to how nature is part of human beings, but also to how humans are perceived as part of nature. The complexity of the relationship is still greater if one considers the standpoint taken by Nisbet and Zelenski (2013, p. 2), who point out that the relationship is “not simply a love of nature, or enjoyment of only the superficially pleasing facets of nature, but rather an awareness and understanding of all aspects of the natural world, even those that are not aesthetically appealing or useful to humans.”

Worth highlighting among the multiple approaches to measuring the relationship between the *self* and nature is the connectedness to nature scale (CNS) developed by Mayer and Frantz (2004) owing to its frequent use (e.g., Brügger et al., 2011; Corraliza and Bethelmy, 2011; Olivos and Aragonés, 2011; Cervinka et al., 2012; Matas-Terrón and Elósegui-Bandera, 2012; Olivos et al., 2013; Olivos and Aragonés, 2014; Zhang et al., 2014). This scale comprises 13 items with a graded response, in which participants must be situated along a continuum of five points. It originally consisted of 14 items. However, both the authors of the original scale and Olivos et al. (2011) noted that item 12 presents factor loadings lower than 0.3, and as a result, it was dropped in later works.

Although the CNS has been frequently used in English and in Spanish and its construct validity has been analyzed, demonstrating its cognitive nature (Perrin and Benassi, 2009), it has never been subjected to an item analysis. Therefore, we considered necessary an analysis to ensure the concept of connectedness was being measured correctly, such as that facilitated by Item Response Theory (IRT) models. These models allow to identify the extent to which each of the items discriminates between individuals with different levels of connectedness, as well as item difficulty parameters, thus providing the response probabilities based on the real level of connectedness. On the other hand, the main characteristic of IRT models is the independence of the sample to which they are applied, which is especially relevant when analyzing a scale used in such diverse contexts. Accordingly, the purpose of the present study is to perform this analysis on the Spanish version of the scale using IRT, and thus refine it to obtain a shorter version with greater accuracy of measurement.

## Study 1

### Method

#### Participants and Instrument

The sample was obtained by requesting the databases of different authors who have used the Spanish-language CNS in their work. The total sample comprised 1008 participants extracted from the databases of Aragonés et al. (2011, 2013) and Olivos et al. (2013). There were no differences between them as regards the means on the CNS. The mean age of the participants was 21.7 (*SD* = 4.2); 74.9% of the participants were female.

#### Data Analysis

The scale was analyzed using IRT models. These models provide estimators of the parameters that characterize the items which do not depend on the samples of participants to whom the test has been applied. Furthermore, the parameters that characterize a participant do not depend on the sample of items employed (Lord, 1980; Hambleton and Swaminathan, 1985). To analyze items such as those of the CNS based on IRT, polytomous models are required to represent the non-linear relationship between the level of the variable and the probability of responding to a given category (Embretson and Reise, 2000). Samejima (1969) proposed graded response models, because responses cannot always be categorized in terms of correctness or error, which is what IRT has traditionally analyzed. This model is especially suitable for items whose response categories are scored between 1 and the number of categories, so the choice of the highest category implies a higher level in the construct, as is the case with CNS.

##### Unidimensionality

One of the fundamental assumptions of unidimensional parametric IRT models is that the measured construct is unidimensional—in other words, that the covariance between items can be explained by a single factor. The CNS scale was originally created to measure the cognitive component of connectedness only. The usual procedure for establishing dimensionality is the use of factor analysis (Embretson and Reise, 2000). To this end, we used the program FACTOR 9.3 (Lorenzo-Seva and Ferrando, 2006), considering the items as ordinals. In addition, this statistical analysis is an aspect that allows validation of the internal structure of the scale (Rios and Wells, 2014).

##### Calibration of the IRT items

To ascertain the quality of each of the items that make up the CNS, we estimated the discrimination and difficulty parameters, through Samejima’s (1969) Graded Response Model (GRM), which estimates individual slopes for each of the CNS items. We evaluated the fit of the model for each item. We carried out IRT analysis with the IRT.PRO program (Cai et al., 2012). Finally, we examined whether there were problems of local dependency. Items exhibiting local dependency will tend to present higher slopes than those shown by other items.

### Results

We first present the descriptive statistics (**Table 1**), which show the means and standard deviations for each of the items, as well as their correlations with the total score on the scale.

**Table 1:** Descriptive statistics of Connectedness to Nature Scale (CNS) items.

	Mean	*SD*	Correlation with total
(1) I often feel a sense of oneness with the natural world around me.	3.57	0.810	0.530^∗^
*A menudo me siento en unión con el mundo natural que me rodea*			
(2) I think of the natural world as a community to which I belong.	3.66	0.828	0.551^∗^
*Pienso en el mundo natural como en la comunidad a la que pertenezco*			
(3) I recognize and appreciate the intelligence of other living organisms.	4.26	0.732	0.411^∗^
*Reconozco y valoro la inteligencia de otros seres vivos*			
(4) I often feel disconnected from nature.	3.06	1.044	0.289^∗^
*Frecuentemente me siento desconectada/o de la naturaleza*			
(5) When I think of my life, I imagine myself to be part of a larger cyclical process of living.	3.34	0.979	0.558^∗^
*Cuando pienso en mi vida me imagino a mí misma/o formando parte de un proceso cíclico más amplio de la vida*			
(6) I often feel a kinship with animals and plants.	3.00	1.079	0.648^∗^
*A menudo me siento emparentada/o con los animales y plantas*			
(7) I feel as though I belong to the Earth as equally as it belongs to me.	3.15	0.973	0.629^∗^
*Siento como si perteneciera a la Tierra de la misma forma que ella me pertenece a mí*			
(8) I have a deep understanding of how my actions affect the natural world.	3.83	0.869	0.486^∗^
*Tengo una intensa comprensión de cómo mis actos afectan al mundo natural*			
(9) I often feel part of the web of life.	3.59	0.843	0.588^∗^
*Frecuentemente me siento parte de la trama de la vida*			
(10) I feel that all inhabitants of Earth, human and non-human, share a common “life force.”	3.52	0.982	0.604^∗^
*Siento que todos los habitantes de la Tierra, humanos y no humanos, comparten una “fuerza vital” común*			
(11) Like a tree can be part of a forest, I feel embedded within the broader natural world.	3.45	0.864	0.667^∗^
*De igual forma que el árbol forma parte del bosque, yo me siento incrustada/o dentro del mundo natural más amplio*			
(12) I often feel like I am only a small part of the natural world around me, and that I am no more important than the grass on the ground or the birds in the trees.	3.21	1.067	0.478^∗^
*A menudo siento que sólo soy una pequeña parte del mundo natural que me rodea, y que no soy más importante que la hierba del suelo o los pájaros de los árboles*			
(13) My personal welfare is independent of the welfare of the natural world.	3.11	1.161	0.304^∗^
*Mi bienestar personal es independiente del bienestar del mundo natural*			

^∗^*p < 0.01*.

#### Evaluation of Dimensionality

Using the Unweighted Least Squares (ULS) method (GFI = 0.98), a single factor was obtained in the Exploratory Factor Analysis, explaining 20.9% of the variance, meaning that unidimensionality was assumed.

#### IRT Calibration

Upon analyzing the overall fit of the scale, we observed a value of -2LL = 31932.26. At item level, the S – χ^2^ statistic was used to assess fit. The results are shown in **Table 2**, in which it can be seen that while most of the items present an acceptable fit, items 4 and 13 do not have a suitable fit in relation to the model (*p* < 0.01). For this reason, these items were eliminated from subsequent analysis.

**Table 2:** S-*X*^2^ fit statistics.

Item	*X*^2^	*df*	Probability
CNS1	76.67	76	0.4577
CNS2	71.91	74	0.5479
CNS3	65.85	68	0.5521
CNS4	235.66	107	0.0001
CNS5	76.16	86	0.7676
CNS6	88.22	84	0.3546
CNS7	107.05	80	0.0234
CNS8	89.18	86	0.3852
CNS9	98.77	76	0.0407
CNS10	93.24	80	0.1476
CNS11	82.86	68	0.1058
CNS12	125.67	96	0.0227
CNS13	150.57	109	0.0052

To determine whether there is local independence (LI) – that is, if there is no additional systematic covariance to the covariance between the underlying construct and the item – LD χ^2^ was calculated, with χ^2^ = 25.1 being found for items 1 and 2. This means that the presence of both items in the scale is redundant since they measure the same aspect of the construct. For this reason, we decided to remove item 1 from the scale, as its discrimination index was lower than that of item 2, and, thus, the latter provides the scale with better psychometric properties. This also occurred with items 7 and 12 (χ^2^ = 10.6); we removed item 12, which had a discrimination index of 0.77.

We then performed a second analysis, in which we included the rest of the items, whose difficulty and discrimination parameters are shown in **Table 3**. First, it can be seen that the values of the discrimination index of item 3 (*a* = 0.80) and item 8 (*a* = 0. 95) do not reach the value 1, and so they do not discriminate particularly well among individuals with different values of connectedness to nature. Both items have negative values in all discrimination parameters except in *b*_4_, so they are items in relation to which it is easy for participants to answer with any of the categories.

**Table 3:** Estimates of the parameters of the items of the graded response model.

Item	Label	*a*	*s.e.*	*b*_1_	*s.e.*	*b*_2_	*s.e.*	*b*_3_	*s.e.*	*b*_4_	*s.e.*
1	CNS2	1.25	0.09	-4.19	0.34	-2.30	0.16	-0.56	0.07	2.08	0.15
2	CNS3	0.80	0.08	-6.78	0.80	-5.18	0.54	-2.75	0.27	0.70	0.11
3	CNS5	1.28	0.09	-2.98	0.20	-1.52	0.11	0.12	0.06	2.12	0.14
4	CNS6	1.33	0.09	-2.09	0.14	-0.66	0.07	0.69	0.07	2.45	0.16
5	CNS7	1.64	0.11	-2.37	0.14	-0.96	0.07	0.46	0.06	2.09	0.13
6	CNS8	0.95	0.08	-4.71	0.43	-2.52	0.21	-1.09	0.11	1.87	0.16
7	CNS9	1.53	0.11	-3.30	0.22	-1.82	0.11	-0.34	0.06	1.95	0.12
8	CNS10	1.60	0.11	-2.61	0.16	-1.53	0.09	-0.17	0.05	1.62	0.10
9	CNS11	1.91	0.13	-2.87	0.17	-1.52	0.08	-0.03	0.05	1.91	0.11

The results obtained upon eliminating these last two items of the scale are shown in **Table 4**. This table shows that the remaining items have high discrimination indices, as well as difficulty indices that grow with the response categories. It is worth noting the quality of item 11 since it yields the highest discrimination parameter and the values for the difficulty parameters increase as the connectedness score rises.

**Table 4:** Estimates of the parameters of the items of the graded response model.

Item	Label	*a*	*s.e.*	*b*_1_	*s.e.*	*b*_2_	*s.e.*	*b*_3_	*s.e.*	*b*_4_	*s.e.*
1	CNS2	1.22	0.09	-4.28	0.35	-2.35	0.16	-0.57	0.07	2.12	0.15
2	CNS5	1.32	0.10	-2.92	0.20	-1.49	0.10	0.12	0.06	2.07	0.14
3	CNS6	1.27	0.09	-2.14	0.14	-0.68	0.07	0.71	0.08	2.52	0.17
4	CNS7	1.62	0.11	-2.38	0.14	-0.97	0.07	0.47	0.06	2.11	0.13
5	CNS9	1.53	0.11	-3.30	0.22	-1.82	0.11	-0.34	0.06	1.95	0.12
6	CNS10	1.63	0.11	-2.58	0.15	-1.51	0.09	-0.16	0.05	1.60	0.10
7	CNS11	1.99	0.14	-2.82	0.16	-1.49	0.08	-0.02	0.05	1.87	0.11

Finally, to see if this modification of the scale reduces the misfit, we calculated the coefficient of determination, which indicates the proportion to which the misfit (Pardo and Ruiz, 2012) has been reduced. We verified that upon eliminating the previous items, the misfit was reduced by 88.63%.

## Study 2

Having shortened the scale by means of the analyses conducted in study 1, it was necessary to verify whether the remaining seven-items of the scale provide adequate reliability and validity. To this end, we conducted the following study.

### Method

#### Participants and Instrument

A total of 321 individuals from the general population of Madrid (Spain) participated in this study. Mean age was 45.42 years (*SD* = 9.62); 53.6% were female. All participants completed a self-administered questionnaire. This consisted of the briefer 7-item version of the CNS; the Love and Care for Nature Scale (LCS) designed by Perkins (2010), which comprises 15 Likert-type items; and the 21-item Nature Relatedness Scale (NR-21) created by Nisbet et al. (2009). Finally, participants provided their sociodemographic data.

Undergraduate students provided a maximum of three questionnaires completed by individuals from different 10-years age groups and of different gender, so as to ensure a heterogeneous sample.

### Results

In order to verify the reliability of the new version of the CNS-7, we conducted a reliability analysis, using Cronbach’s alpha, which yielded adequate reliability (α = 0.866). Furthermore, to analyze the convergent validity, we calculated the correlations between the CNS-7 and the LCS (*r* = 0.63, *p* < 0.01, *n* = 321) and NR-21 (*r* = 0.60, *p* < 0.01, *n* = 321).

## Discussion

The need for a comprehensive analysis of the CNS is based on its widespread use in the field of studies in connection with nature. This analysis of the Spanish version attempts to determine whether the construct is correctly measured. The analyses carried out in this work show that the misfit that occurs in relation to the full 13-item scale is greatly reduced in the case of the shorter scale, following the removal of six items. First, items 4 (“I often feel disconnected from nature”) and 13 (“My personal welfare is independent of the welfare of the natural world”) were removed due to lack of fit. It is worth reflecting on item 4, since its wording makes explicit reference to the degree to which people feel connected to nature. If researchers use this item when they do not need a precise measure of connectedness, its use could lead to erroneous conclusions, since it does not present a good fit when measuring connectedness.

Second, we found redundant items in the scale, due to local dependency between items 1 (“I often feel a sense of oneness with the natural world around me”) and 2 (“I think of the natural world as a community to which I belong”), and between items 7 (“I feel as though I belong to the Earth as equally as it belongs to me”) and 12 (“I often feel like I am only a small part of the natural world around me, and that I am no more important than the grass on the ground or the birds in the trees”). Moreover, we noted that both item 3 (“I recognize and appreciate the intelligence of other living organisms”) and item 8 (“I have a deep understanding of how my actions affect the natural world”) do not discriminate well between individuals with different levels of connectedness. That is, the fact that an individual greatly recognizes and values the intelligence of other living beings does not necessarily imply that he or she is connected with nature. However, the remaining items had both a good fit and adequate discrimination and difficulty indices.

Furthermore, upon analyzing the new version of the scale in Study 2, we found that its reliability is high and, indeed, slightly higher than that obtained by Mayer and Frantz (2004) for their original 14-item scale. The convergent validity established with the LCS and NR-21 scales are also high if we consider the correlations obtained by Mayer and Frantz with the New Environmental Paradigm (NEP) scale and the biospheric value orientation, or those obtained by Olivos et al. (2011) with the Environmental Identity and Inclusion of Nature of Self scales.

By reducing the scale to seven items, we propose it would correctly measure connectedness insofar as the scores obtained would actually discriminate between individuals who are connected and those who are not, since they would have response probabilities to one category or another in relation to their actual level of connectedness. Thus, it can be concluded that some items from the original scale add noise to the measurement and that, as well as being more reliable, the new 7-item version is easier to administer.

In addition, we consider it important to point out the quality of item 11 (“Like a tree can be part of a forest, I feel embedded within the broader natural world”). This item proved to be very informative, and as a result it may be the best option in the event that it is necessary to measure connectedness with a single item.

### Future Direction

The Spanish version of the CNS (Olivos et al., 2011) seems to be a suitable option for measuring this concept provided that it is carried out using the seven items—2, 5, 6, 7, 9, 10, and 11—that presented good psychometric properties. In light of the results obtained, it would be appropriate to perform an analysis of the original scale (Mayer and Frantz, 2004) with English-speaking samples to verify to what degree the findings are similar to those contained in this work.

Furthermore, attention should be paid to what is understood by nature since, as Duffy and Verges (2009) suggest, individual differences in connectedness could be influenced by how people conceive of it.

## Author Contributions

LP, JA, and MC conceived and designed the work. LP analyzed the data. LP, JA, and MC wrote the paper.

## Conflict of Interest Statement

The authors declare that the research was conducted in the absence of any commercial or financial relationships that could be construed as a potential conflict of interest.
